# In vitro characterisation of [^177^Lu]Lu-DOTA-C595 as a novel radioimmunotherapy for MUC1-CE positive pancreatic cancer

**DOI:** 10.1186/s41181-023-00204-4

**Published:** 2023-08-14

**Authors:** Ashleigh Hull, William Hsieh, William Tieu, Dylan Bartholomeusz, Yanrui Li, Eva Bezak

**Affiliations:** 1 Allied Health and Human Performance Academic Unit, University of South Australia, City East Campus, Cnr North Tce and Frome Road, Adelaide, SA 5001 Australia; 2 Department of PET, Nuclear Medicine and Bone Densitometry, Royal Adelaide Hospital, SA Medical Imaging, Adelaide, SA 5000 Australia; 3 School of Physical Sciences, The University of Adelaide, Adelaide, SA 5000 Australia; 4 Adelaide Medical School, The University of Adelaide, Adelaide, SA 5000 Australia

**Keywords:** Lutetium-177, Radioimmunotherapy, Pancreatic cancer, MUC1, C595

## Abstract

**Background:**

Pancreatic ductal adenocarcinoma (PDAC) continues to be a malignancy with an unmet clinical demand. Development of radioimmunoconjugates which target cancer-specific receptors provides an opportunity for radioimmunotherapy of both metastatic and primary PDAC. In this study, we characterised the in vitro behaviour of a novel beta-emitting radioimmunoconjugate [^177^Lu]Lu-DOTA-C595 as a therapeutic agent against PDAC. [^177^Lu]Lu-DOTA-C595 is designed to target cancer-specific mucin 1 epitopes (MUC1-CE) overexpressed on most epithelial cancers, including PDAC.

**Results:**

A series of in vitro experiments were performed on PDAC cell lines (PANC-1, CAPAN-1, BxPC-3 and AsPC-1) exhibiting strong to weak MUC1-CE expression. [^177^Lu]Lu-DOTA-C595 bound to all cell lines relative to their expression of MUC1-CE. [^177^Lu]Lu-DOTA-C595 was also rapidly internalised across all cell lines, with a maximum of 75.4% of activity internalised within the PANC-1 cell line at 48 h. The expression of γH2AX foci and clonogenic survival of PANC-1 and AsPC-1 cell lines after exposure to [^177^Lu]Lu-DOTA-C595 were used to quantify the in vitro cytotoxicity of [^177^Lu]Lu-DOTA-C595. At 1 h post treatment, the expression of γH2AX foci exceeded 97% in both cell lines. The expression of γH2AX foci continued to increase in PANC-1 cells at 24 h, although expression reduced in AsPC-1. Clonogenic assays showed a high level of cell kill induced by [^177^Lu]Lu-DOTA-C595.

**Conclusion:**

[^177^Lu]Lu-DOTA-C595 has favourable in vitro characteristics to target and treat MUC1-CE positive PDAC. Further investigations to characterise the in vivo effects and potential value of [^177^Lu]Lu-DOTA-C595 in other MUC1-CE expressing malignancies such as lung, ovarian and colorectal adenocarcinoma are warranted.

## Background

Despite ongoing advances in diagnosis and therapy, pancreatic ductal adenocarcinoma (PDAC) continues to be a leading cause of cancer-related death. The 2020 GLOBCAN estimates the incidence and mortality of PDAC were nearly identical and contributed to 495,773 new cases of cancer and 466,003 of cancer-related death worldwide (Sung et al. 2021). Surgical resection remains the only curative treatment of PDAC, however only a small percentage of patients present with resectable or borderline resectable disease (Klaiber et al. 2021; Park et al. 2021; Bockhorn et al. 2014). For most patients presenting with locally advanced or metastatic disease, first-line therapy relies on FOLFIRONOX or gemcitabine-plus albumin-bound (nab) paclitaxel chemotherapy. While chemotherapy can prolong patient survival (Conroy et al. 2011; van der Sijde et al. 2022; Suker et al. 2016), the overall outcomes for patients diagnosed with PDAC remain poor with a 5-year survival rate of only 11.5% (Wood et al. 2022; Thibodeau and Voutsadakis 2018; SEER 2022). Improvements in PDAC survival rely on systemic and personalised therapies capable of targeting distant metastases.

Mucin 1 (MUC1) is a transmembrane glycoprotein that is a receptor-of-interest for targeted therapies against PDAC (Sorbara et al. 2022; Taylor-Papadimitriou et al. 2018; Roulois et al. 2013). MUC1 is normally expressed on the apical surface of epithelial cells and has a role in cell signalling and anti-adhesion properties. In epithelial malignancies such as PDAC, a dysregulation of the glycosylation pattern of normal MUC1 reveals novel cancer-specific epitopes (MUC1-CE) (Constantinou et al. 2011; Gendler et al. 1988; Chen et al. 2021). Several studies have shown that MUC1-CE is expressed in over 90% of pancreatic cancers (Qu et al. 2004; Hull et al. 2020a). Expression of MUC1-CE has also been linked with chemoresistance and disease progression (Tinder et al. 2008; Jonckheere et al. 2010; Suh et al. 2017), thus targeting of MUC1-CE positive pancreatic cancer cells could have value in treating advanced PDAC. Furthermore, anti-MUC1-CE therapies could be applied to other epithelial cancers including ovarian, colorectal and lung adenocarcinoma where MUC1-CE is also overexpressed (Chen et al. 2021).

A wide range of antibodies have been developed to target MUC1-CE including PAM4, Gatipotuzumab, TAB004, MA5 and C595 (Danielczyk et al. 2006; Gold et al. 2013; Wu et al. 2018a; Price et al. 1990). Radiolabelling of these antibodies to a therapeutic radionuclide which emits alpha particles, beta particles or auger electrons has been shown to elicit a cytotoxic effect (Hull et al. 2020b). This radioimmunotherapy (RIT) approach allows for systemic yet localised targeting of MUC1-CE expressing cells which can be harnessed to treat metastatic disease including micro-metastases.

We have previously developed ^177^Lutetium-DOTA-C595 ([^177^Lu]Lu-DOTA-C595) as a radioimmunoconjugate against PDAC expressing MUC1-CE (Hull et al. 2022). C595 is an IgG3 mouse monoclonal antibody raised against the Arg-Pro Ala-Pro epitope of MUC1-CE (Gendler et al. 1988; Price et al. 1990). Over 90% of PDAC cells express C595-reactive MUC1 and it is minimally expressed on normal epithelial cells (Qu et al. 2004; Hull et al. 2020a). C595 may have a future role in PDAC theranostics and has previously been labelled to both therapeutic and diagnostic radionuclides with success, including Bismuth-213 (Qu et al. 2004, 2005; Song et al. 2008), Rhenium-188 (Murray et al. 2001), Copper-67 (Hughes et al. 1997), Copper-64 (Hull et al. 2022) and Technetium-99m (Simms et al. 2001). As a beta-emitting radionuclide, Lutetium-177 (Lu-177) has established itself within the clinical nuclear medicine field due to its increasing availability, 6.7 day half-life allowing for optimal tumour diffusion before decay, and medium range beta-emissions which provide cross-fire effects to target solid tumours. Lu-177 has shown exceptional value as a therapeutic radionuclide in prostate cancer and neuroendocrine tumour therapies, with an acceptable level of side-effects (Strosberg et al. 2017; Sartor et al. 2021).

It has been shown that [^177^Lu]Lu-DOTA-C595 binds to MUC1-CE positive pancreatic cancer cells (Hull et al. 2022). The in vitro kinetics beyond cellular binding of [^177^Lu]Lu-DOTA-C595 are unknown. The aim of this study was to evaluate the in vitro characteristics of [^177^Lu]Lu-DOTA-C595 across pancreatic cancer cell lines with different expression of MUC1-CE to establish its feasibility as RIT for PDAC.

## Methods

### Chelators, antibodies, radionuclides

The bifunctional chelator, p-SCN-Bn-DOTA, was purchased from Macrocyclics, USA. The C595 antibody was purchased from QED Bioscience, USA. Lu-177 was purchased from ANSTO, Australia.

### Conjugation of p-SCN-Bn-DOTA to C595 antibody

A 40-fold molar excess of p-SCN-Bn-DOTA was incubated with C595 antibody for 2 h in a 0.1 M sodium bicarbonate buffer (pH 8.5) at 37 °C in a ThermoMixer® (Eppendorf, Germany) at 700 RPM. Unbound DOTA was removed through a purification process using 50 kDa Amicon® centrifugal filter units. The conjugation process was confirmed by performing concurrent bicinchoninic acid assay (BCA) and Cu(II)-Arsenazo(III) assays to quantify the ratio of C595 molecules (protein concentration via BCA) to bound DOTA groups (Cu(II)-Arsenazo(III) assay] as described by Al-Ejeh et al. (2009). All conjugations resulted in an average DOTA to C595 ratio of 3:1–4:1. The final DOTA-C595 conjugate was buffer transferred into 0.1 M ammonium acetate at pH 6 and stored at 4 °C until required for radiolabelling.

### Radiolabeling of DOTA-C595

Initially 95 MBq of Lu-177 (ANSTO, Australia) was added to a vial containing 100 µL of 0.5 M ammonium acetate (pH 6.5–7). One milligram of DOTA-C595 was added to the vial and the reaction vial was placed in a Thermomixer set to 37 °C, 550 RPM shaking for 1 h. Following incubation, the reaction was purified using 10 kDa Amicon tubes. Three washes with DPBS were performed. The final radioimmunoconjugate was assessed using radio-TLC. Radiochemical yield was determined to be 92.35%. Considering the radiochemical yield, the final specific activity was calculated to be 88 kBq/µg. All assays were performed using the same specific activity. Stability testing of [^177^Lu]Lu-DOTA-C595 has previously been performed (Hull et al. 2022).

### Cell lines

Four human pancreatic cancer cell lines (PANC-1, CAPAN-1, BxPC-3 and AsPC-1) were purchased from American Type Culture Collection (ATCC) (Manassas, USA) via In Vitro Technologies Pty Ltd (Noble Park North, Australia). The cell lines were selected due to their differential expression of MUC1-CE as previously investigated (Hull et al. 2020a). The percentage of positive MUC1-CE cells were as follows: 93.1% (PANC-1), 75.3% (CAPAN-1), 17.3% (BxPC-3) and 11.3% (AsPC-1) (Hull et al. 2020a). PANC-1 cells were cultured in Dulbecco’s Modified Eagle Medium supplemented with 10% of foetal calf serum (FCS) and 1% penicillin/streptomycin (P/S). CAPAN-1 cells were cultured in Iscove’s Modified Dulbecco’s Medium supplemented with 20% FCS and 1% P/S. BxPC-3 and AsPC-1 cells were cultured using Roswell Park Memorial Institute 1640 medium supplemented with 10% FCS and 1% P/S. For all experiments, cells were grown to confluence in T75 flasks in an incubator set to 37 °C and consisting of a 5% carbon dioxide in air environment. At confluence, cells were washed twice with DPBS and detached from the flasks using by TrypLE™ Select Enzyme (1×) (Thermo Fisher Scientific Australia Pty Ltd, Scoresby, Australia). All cell lines were used within 3 months of resuscitation.

### Cell binding

Briefly, 1 × 10^5^ cells per well were seeded into 24-well plates and incubated overnight at 37 °C to allow cell attachment. Cells were replenished with 250 µL of fresh media the following day. Concentrations of [^177^Lu]Lu-DOTA-C595 (0–400 nM, 0.033–1.32 MBq) were added to cells in triplicate samples and incubated for 1 h at 37 °C. Following incubation, excess media was removed and cells were washed twice with cold DPBS. Cells were detached from the well plate using trypsin and cell samples were counted in a WIZARD^2^ automatic gamma counter (PerkinElmer, USA). The average counts per minute for each cell line were plotted against [^177^Lu]Lu-DOTA-C595 concentration and fitted with simple linear regression. A two-way ANOVA with post-hoc Tukey’s test was performed to evaluate for differences in the binding of the cell lines at different concentrations. The average cell counts across all concentrations were correlated with the percentage of MUC1-CE positive cell lines as determined and reported in our previous publication (Hull et al. 2020a) using simple linear regression.

### Internalisation assay

To evaluate the rate of cellular internalisation of [^177^Lu]Lu-DOTA-C595 across different MUC1-CE expressing cell lines, an internalisation assay was performed over 48 h. Initially 5 × 10^5^ cells were seeded into 24-well plates and incubated overnight at 37 °C to allow cell attachment. The following day, cells were replenished with 250 µL of fresh media. [^177^Lu]Lu-DOTA-C595 (200 nM, 0.066 MBq) was then added to the cells. Non-irradiated cells treated with DPBS were used as controls. All cells were incubated at 37 °C in triplicate samples. At t = 1, 18, 24 and 48 h, unbound activity was removed via the supernatant and cells were washed twice with DPBS. Surface-bound activity was removed by incubating cells with 200 µL of 0.2 M acetic acid in 0.5 M sodium chloride for 5 min at 37 °C. Cells were lysed with 0.1 M sodium hydroxide for 10 min and washed with 200 µL of TryPLE Select to remove all cell lysate. Both surface-bound and cell lysate fractions were counted as triplicate samples in the WIZARD^2^ automatic gamma counter. The counts of each sample were background- and decay-corrected and plotted over time as four separate graphs: surface-bound, internalised, total activity (sum of surface-bound and internalised counts) and the percentage of internalisation (internalised fraction divided by total activity). A two-way ANOVA with post-hoc Tukey’s test was performed to evaluate differences in the percentage of internalisation of [^177^Lu]Lu-DOTA-C595 in the cell lines at each time point.

### γH2AX foci

Staining of γH2AX foci was used as a marker for detecting double strand DNA breaks. Initially, 5 × 10^5^ AsPC-1 and PANC-1 cells were adhered to a 24-well plate overnight. [^177^Lu]Lu-DOTA-C595 (1000 nM, 3.3 MBq) was added to the cells and incubated for 2 h at 37 °C. Following incubation, cells were washed with DPBS to remove unbound activity and replenished with fresh media. At t = 1 and 24 h post wash, both treated and untreated control cells were fixed and permeabilised using ice-cold methanol then stored at 4 °C. Cells were resuspended in FACS buffer consisting of 1% bovine serum albumin in PBS then seeded at 2 × 10^5^ cells per flow tube. Cells were then stained using either Alexa Fluor 488 Phospho-Hist99one H2A.X (Ser139) Monoclonal Antibody (CR55T33) or Mouse IgG1 kappa Isotype Control (P3.6.2.8.1) and Alexa Fluor 488, eBioscience (ThermoFisher 53-4714-80) at 5 µg/mL. Samples were analysed using a Cytoflex S Flow Cytometer (Beckman Coulter, USA). Cells stained with the isotype control and unstained cell samples were used as gating controls. At least 10,000 events were analysed per sample. The percentage of positive γH2AX cells was averaged for each time point and cell line. T-tests were used to evaluate for statistical significance between the mean percentage of cells with positive γH2AX expression at each time point and between cell lines.

### Clonogenic assay

To evaluate the cytotoxic effects of [^177^Lu]Lu-DOTA-C595, a clonogenic assay was performed using PANC-1 and AsPC-1 cell lines. Cells (2 × 10^5^) were seeded into 24-well plates and incubated overnight at 37 °C. The following day, samples were incubated with [^177^Lu]Lu-DOTA-C595 (100, 250, 500, 750, 1000, 2000 and 3000 nM, 0.33–9.9 MBq) for 24 h in an incubator set to 37 °C. Additional samples were treated with free Lu-177, unlabeled DOTA-C595, unmodified C595 or used as untreated controls. Following incubation, cells were seeded into 6-well plates at different cell densities depending on treatment condition. Triplicate samples were seeded for each condition and cells were replenished with 2 mL of fresh media. Cells were monitored for growth with media replenished every 2–3 days, until a defined end point of 14 days. At the end-point, colonies were stained using 50% methanol:crystal violet. Colonies were manually counted using a transparent grid on a ZEISS inverted microscope (Oberkochen, Germany). The colony threshold was set to a minimum of 50 cells. To determine intra-observer reliability, 24 wells were recounted following a 1 month break and the absolute intra-class correlation coefficient was calculated using IBM® SPSS® Statistics (v. 28.0.1.1 Constantinou et al. 2011, New York, USA).

The plating efficiency (PE) was calculated for each cell line using the untreated controls and was defined as the number of colonies divided by the number of seeded cells (Eq. 1). The surviving fraction (SF) of treated cells was calculated as the number of colonies divided by the number of seeded cells and normalised by the PE of the relevant cell line (Eq. 2), as follows:

1 $$PE = \left( {\frac{Number\;of\;colonies}{{Number\;of\;seeded\;cells}}} \right) \times 100$$

2 $$SF = \left( {\frac{Number\;of\;colonies}{{Number\;of\;seeded\;cells \times PE}}} \right) \times 100$$

### Statistics

All statistical analyses were performed using GraphPad Prism (v.9.2.0, GraphPad Prism Software, USA) unless otherwise stated. Statistical significance was defined at *p* < 0.05 unless otherwise stated.

### Ethics

This study was approved by the University of South Australia’s Human Research Ethics Committee (protocol number: 202278).

## Results

### Cell binding

**Fig. 1:**
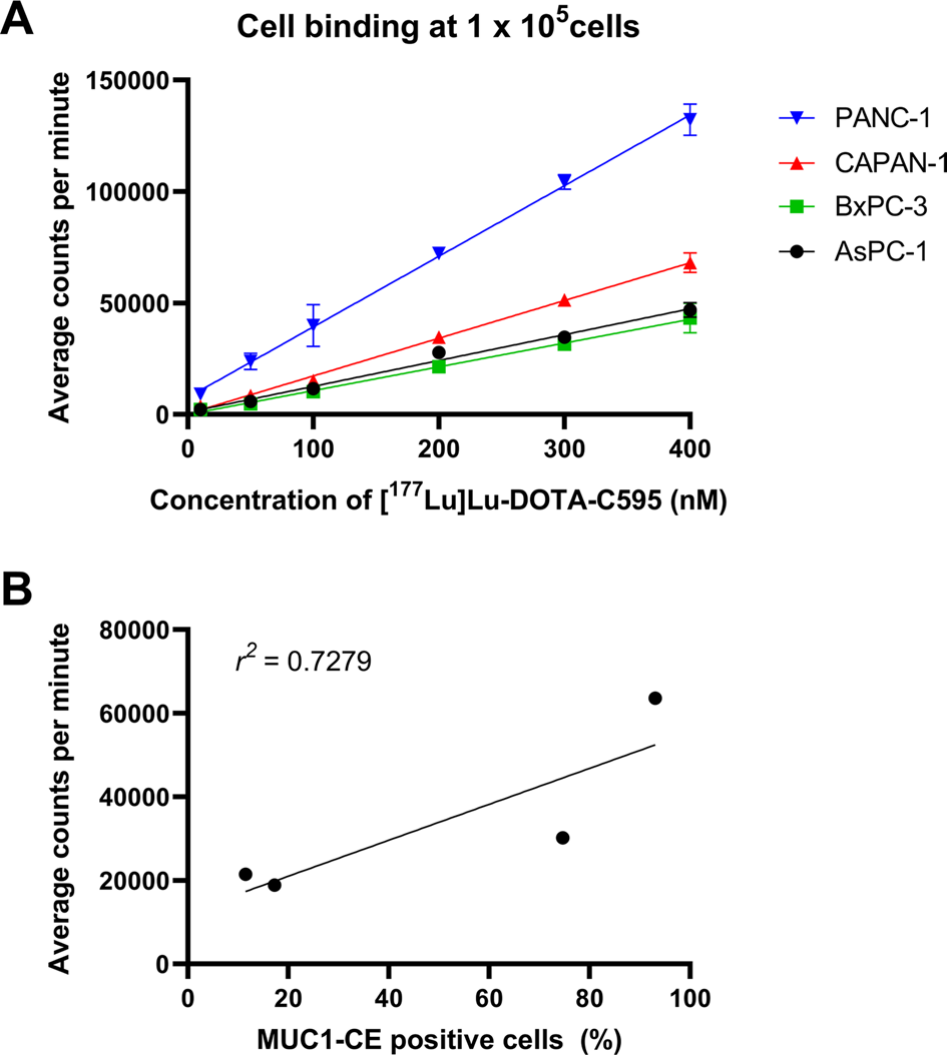
**A** Cell binding of [^177^Lu]Lu-DOTA-C595 at 1 × 10^5^ cells corrected for background and **B** linear regression of the percentage of positive MUC1-CE cells and average cell binding counts per minute

All cell lines demonstrated a linear relationship between the concentration of [^177^Lu]Lu-DOTA-C595 added and bound radioactivity (*r*^*2*^ values of 0.9915, 0.9927, 0.9724 and 0.9828 respectively for PANC-1, CAPAN-1, BxPC-3 and AsPC-1 cells) (Fig. 1A). Binding of [^177^Lu]Lu-DOTA-C595 was significantly greater to PANC-1 cells than all other cell lines at concentrations of 50, 200, 300 and 400 nM. Binding to CAPAN-1 cell lines was also significantly greater than to BxPC-3 and AsPC-1 cell lines at concentrations of 200 nM and above.

A moderate correlation (*r*^*2*^ = 0.7279) was identified between the percentage of MUC1-CE positive cells and the average counts per minute from the cell binding experiment at cell concentrations of 1 × 10^5^ (Fig. 1B).

### Internalisation assay

**Fig. 2:**
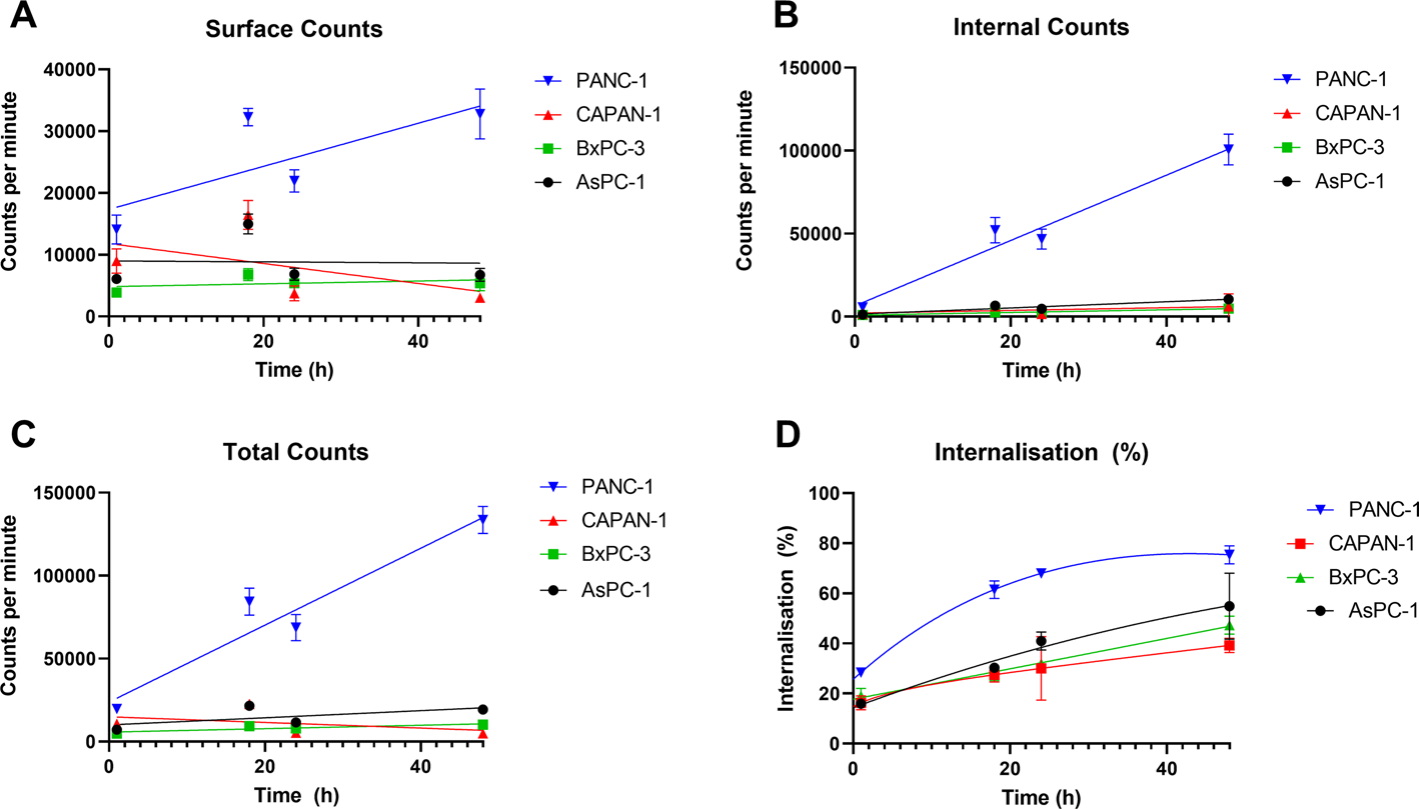
Background- and decay-corrected cell counts across 48 h representing **A** surface binding, **B** internalised fraction, **C** total binding including surface and internalised and **D** rate of internalisation

The surface binding of [^177^Lu]Lu-DOTA-C595 was inconsistent across the cell lines (Fig. 2A). PANC-1 cells were the only cell line where surface binding increased as time progressed. In comparison, BxPC-3 and AsPC-1 cells demonstrated similar surface binding counts at each time point, suggesting early saturation of available MUC1-CE receptors. CAPAN-1 cells showed a decrease in surface binding over time which is likely attributed to cell sloughing, a known characteristic of CAPAN-1 cells (ATCC 2022).

The internalised fraction of activity demonstrated rapid increases in PANC-1 cells as time progressed, whilst all other cell lines demonstrated lower levels of internalisation with only mild increases in internalised counts over time (Fig. 2B).

All four cell lines demonstrated a steady rate of internalisation which increased with time (Fig. 2D and Table 1). PANC-1 cell lines had the greatest percentage of internalisation at all time points, beginning with 28.4% at 1 h and progressing to a maximum of 75.4% at 48 h. A similar trend was noted for the other cell lines, although internalisation was not as rapid as that for PANC-1. The internalised percentage of activity was significantly greater in PANC-1 cells than in all other cell lines at 1 and 18 h. BxPC-3 and AsPC-1 cells also had a significantly lower internalisation rate than PANC-1 at 24 h, which also continued to 48 h for BxPC-3. CAPAN-1 also demonstrated significantly lower internalisation percentage at 48 h compared to PANC-1 cells. No other significant differences were identified between the cell lines.

**Table 1:** Rate of cellular internalisation of [^177^Lu]Lu-DOTA-C595 over 48 h

Cell line	Mean internalised activity ± S.D. (%) *p* value compared to PANC-1			
	1 h	18 h	24 h	48 h
PANC-1	28.415 ± 1.833	61.499 ± 3.572	67.983 ± 1.155	75.370 ± 3.554
CAPAN-1	16.295 ± 2.773 *p* = 0.0162*	27.492 ± 2.905 *p* = 0.0009***	30.036 ± 12.683 *p* = 0.0848	39.201 ± 2.803 *p* = 0.0007***
BxPC-3	19.025 ± 3.054 *p* = 0.0492*	27.776 ± 2.611 *p* = 0.0011**	31.968 ± 1.736 *p* < 0.0001****	47.312 ± 3.629 *p* = 0.0023**
AsPC-1	15.927 ± 1.916 *p* = 0.0045**	30.332 ± 1.740 *p* = 0.0031**	40.933 ± 3.593 *p* = 0.0084**	54.872 ± 13.186 *p* = 0.2588

Displayed *p*-values were computed using Tukey’s Test and compared to PANC-1 cells at corresponding time point

^*^p < 0.05, **p < 0.01, ***p < 0.001 and ****p < 0.0001

### Induction of γH2AX foci

**Fig. 3:**
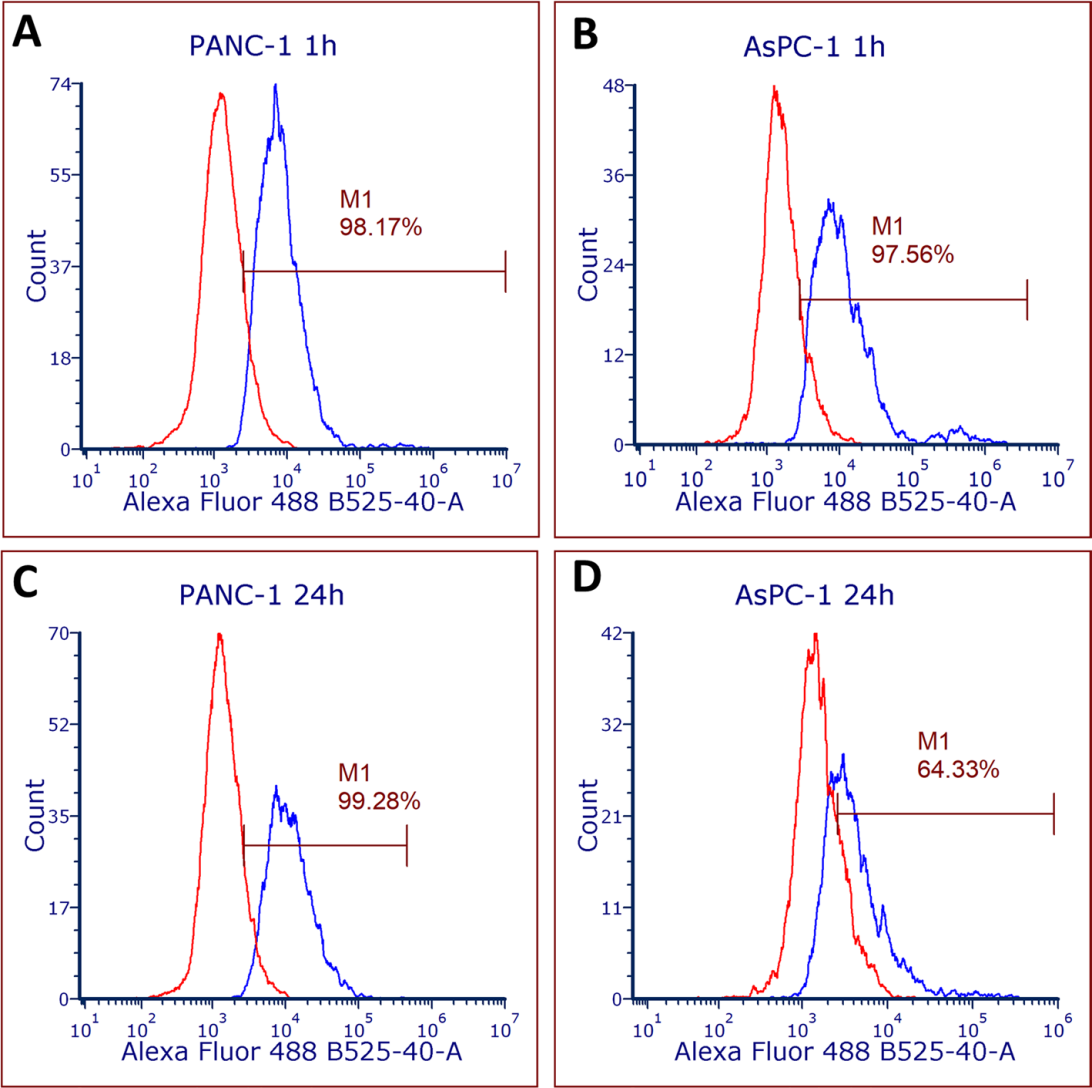
Representative flow cytometry histograms of γH2AX expression in PANC-1 and AsPC-1 cells at **A** and **B** 1 h, and **C** and **D** 24 h following [^177^Lu]Lu-DOTA-C595 treatment

At 1 h post-treatment, the percentage of cells with γH2AX detected was similar between PANC-1 and AsPC-1 cells at 98.56 and 97.01%, respectively (*p* = 0.263618). At 24 h post-treatment, 99.35% of PANC-1 cells exhibited positive γH2AX expression. There was no significant difference identified in the expression of γH2AX on PANC-1 cells between the 1 h and 24 h post-treatment groups (*p* = 0.264814). AsPC-1 cells demonstrated lower γH2AX expression of 69.2% at 24 h which significantly differed from the PANC-1 cells (*p* = 0.008651) and the 1 h AsPC-1 cells (*p* = 0.011965) (Fig. 3). This may suggest resolution of double-strand DNA breaks in the AsPC-1 cells within 24 h.

### Clonogenic assay

**Fig. 4:**
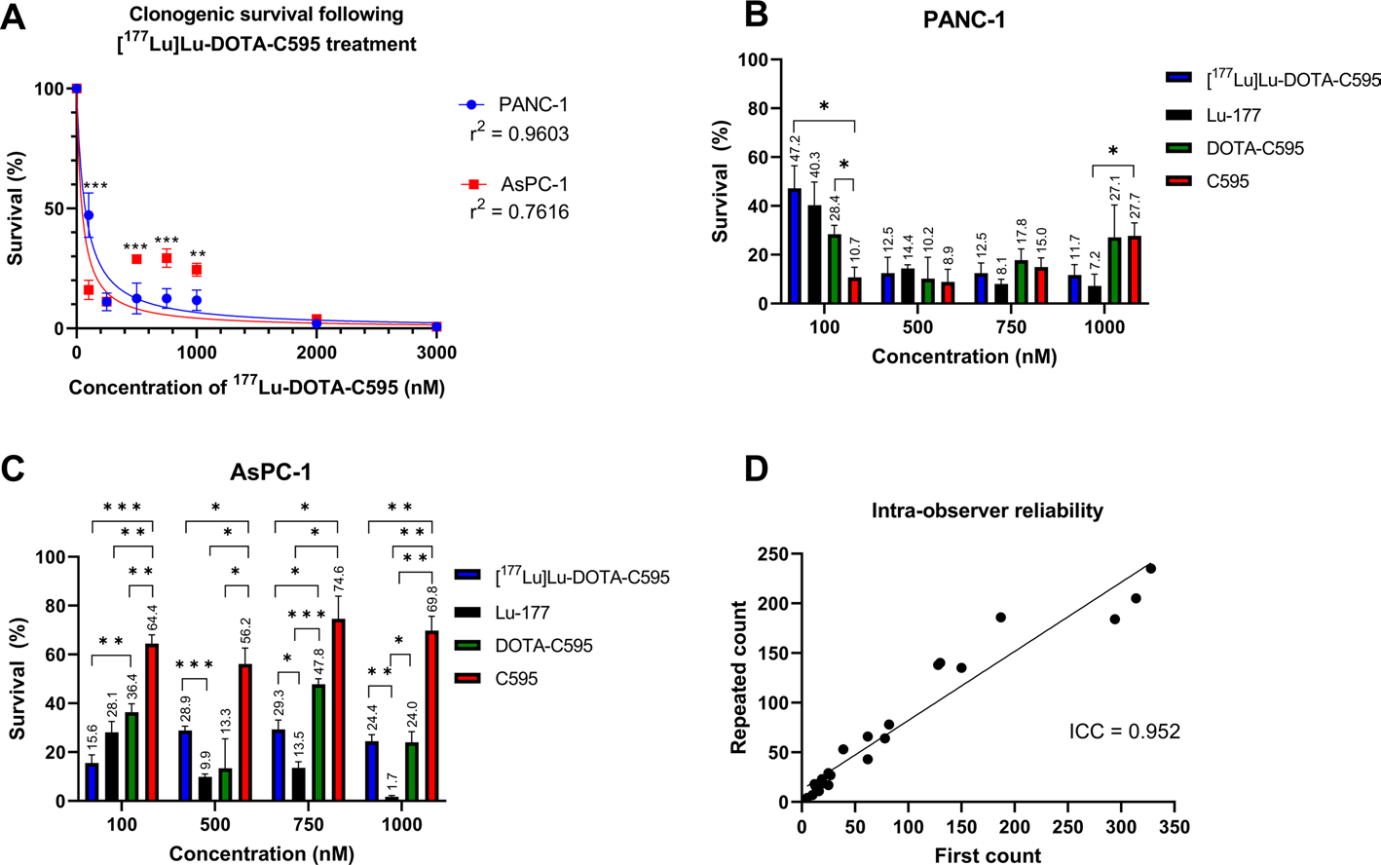
Clonogenic survival of **A** PANC-1 and AsPC-1 at varying concentrations of [^177^Lu]Lu-DOTA-C595, **B** PANC-1 and **C** AsPC-1 cell survival following exposure to equivalent concentrations of [^177^Lu]Lu-DOTA-C595, Lu-177, DOTA-C595 and C595 and **D** intra-observer reliability of manual counting determined using the intra-class correlation coefficient (ICC). Data are presented as mean ± standard deviation. *p < 0.05, **p < 0.01, ***p < 0.001

The clonogenic survival of PANC-1 and AsPC-1 cells varied with treatment type and concentration (Table 2). [^177^Lu]Lu-DOTA-C595 exhibited a cytotoxic effect on both AsPC-1 and PANC-1 cell lines at all concentrations (Fig. 4A). When compared between cell lines, PANC-1 had significantly less clonogenic survival at concentrations of 500, 750 and 1000 nM compared to AsPC-1 cells (*p* < 0.001, *p* < 0.001 and *p* = 0.0016, respectively). However, at the 100 nM concentration, PANC-1 cells had significantly greater survival than AsPC-1 cells (*p* < 0.001). No significant differences were identified at the 250, 2000 and 3000 nM concentrations.

**Table 2:** Clonogenic survival between cell lines and treatment types at different concentrations

Concentration (nM)	Mean survival ± S.D. (%)							
	PANC-1				AsPC-1			
	[^177^Lu]Lu-DOTA-C595	Lu-177	DOTA-C595	C595	[^177^Lu]Lu-DOTA-C595	Lu-177	DOTA-C595	C595
100	47.2 ± 9.3	40.3 ± 9.5	28.4 ± 3.7	10.7 ± 4.1	15.6 ± 3.3	28.1 ± 4.4	36.4 ± 3.4	64.4 ± 3.6
250	11.0 ± 3.7	–	–	–	11.1 ± 1.3	–	–	–
500	12.5 ± 6.4	14.4 ± 1.5	10.2 ± 8.8	8.90 ± 5.1	28.9 ± 1.7	9.90 ± 1.1	13.3 ± 12.1	56.2 ± 6.5
750	12.5 ± 4.1	8.05 ± 1.9	17.8 ± 4.6	15.0 ± 3.7	29.3 ± 3.9	13.5 ± 2.5	47.8 ± 2.3	74.6 ± 9.3
1000	11.7 ± 4.2	7.20 ± 4.8	27.1 ± 13.2	27.7 ± 5.4	24.4 ± 2.7	1.74 ± 0.5	24.1 ± 4.3	69.8 ± 5.8
2000	1.98 ± 0.98	–	–	–	3.85 ± 0.78	–	–	–
3000	0.74 ± 1.0	–	45.8 ± 24.8	19.6 ± 6.6	0.72 ± 0.74	–	43.5 ± 14.8	50.6 ± 1.8

Only three statistically significant differences were identified in the survival of PANC-1 cells following [^177^Lu]Lu-DOTA-C595, Lu-177 only, DOTA-C595 only and C595 only treatments at equivalent concentrations (Fig. 4B). At 100 nM, C595 treated cells had the lowest surviving fraction which significantly differed from cells treated with [^177^Lu]Lu-DOTA-C595 (*p* = 0.0310) and DOTA-C595 (*p* = 0.0181). Cell survival was low (less than 22%) yet comparable between all treatment conditions at 500 and 750 nM. At 1000 nM, cells treated with Lu-177 had significantly lower survival than those treated with C595 (*p* = 0.0273). Survival of PANC-1 cells treated with [^177^Lu]Lu-DOTA-C595 or Lu-177 appeared similar across all concentrations.

AsPC-1 cells treated with unlabeled C595 had significantly greater survival than all other treated AsPC-1 cells at all concentrations, except for 750 nM of DOTA-C595 (Fig. 4C). [^177^Lu]Lu-DOTA-C595 treated cells had lower surviving fractions compared to those treated with DOTA-C595 at concentrations of 100 and 750 nM (*p* = 0.0058 and* p* = 0.0136, respectively). Significant differences were also identified between the survival of cells treated with 750 and 1000 nM of DOTA-C595 and equivalent concentrations of Lu-177 treated cells (*p* = 0.0002 and* p* = 0.0289, respectively). Interestingly, AsPC-1 cells treated with 500, 750 and 1000 nM of Lu-177 had significantly lower survival than those treated with equivalent concentrations of [^177^Lu]Lu-DOTA-C595 (*p* = 0.0008, *p* = 0.0210 and *p* = 0.0097, respectively).

The intra-class correlation coefficient (ICC) was determined to be 0.952, indicating strong intra-observer reliability in the manual colony counting process (Fig. 4D).

## Discussion

PDAC is an aggressive solid tumour often diagnosed at a late stage when prognosis is poor. Current treatment of PDAC is often limited or ineffective against widespread metastases. RIT may provide additive or synergistic effects to current clinical treatments and improve targeting of advanced PDAC. This study further characterised the in vitro behavior of [^177^Lu]Lu-DOTA-C595 as a possible RIT against PDAC.

Specific binding to the target receptor is a cornerstone of any antibody-based therapy, however it is an inherent requirement of RIT where off-targeted effects could be detrimental to normal cells (Zhao et al. 2020). We have previously shown that [^177^Lu]Lu-DOTA-C595 binds to two PDAC cells in relation to their MUC1-CE expression (Hull et al. 2022). In this study, we aimed to confirm this finding by expanding the cellular binding assay to four PDAC cells with differing MUC1-CE expression. Strong binding was identified across the cell lines in an expected pattern relative to their MUC1-CE expression. Cells with low MUC1-CE expression still effectively bound low levels of [^177^Lu]Lu-DOTA-C595, suggesting a therapeutic effect may be possible across PDAC cells with varying levels of MUC1-CE expression. The prognosis of PDAC has been shown to vary with MUC1-CE expression (Striefler et al. 2021); thus having the capability to target both high- and low-MUC1-CE expressing cells in a single treatment could be beneficial in a clinical setting. Given MUC1-CE expression is believed to increase as PDAC develops and progresses, targeting of cells with low MUC1-CE expression may provide an opportunity to treat PDAC cells earlier in their development (Rachagani et al. 2012).

Cellular internalisation is a key mechanism of radioimmunoconjugate uptake and can influence the overall toxicity of the therapy. For low energy beta emitters such as Lu-177, a higher rate of internalisation may increase damage to the DNA of the targeted cells as the likelihood of ionisation is increased due to the shorter distance between the decay event and nuclear DNA (Tamborino et al. 2020). In this study, we aimed to establish the rate of internalisation of [^177^Lu]Lu-DOTA-C595 across the different PDAC cell lines. While internalisation was highest in PANC-1 cells, AsPC-1 cells also demonstrated high rates of [^177^Lu]Lu-DOTA-C595 internalisation despite low MUC1-CE expression. BxPC-3 and CAPAN-1 cells exhibited lower rates of internalisation. Although cell surface binding is a key factor governing internalisation, the efficiency of internalisation can be highly cell dependent and heterogeneous across similar cell populations, particularly for large macromolecules such as monoclonal antibodies (Kuo et al. 2009). The difference in cell line behaviour may explain the high internalisation of [^177^Lu]Lu-DOTA-C595 in AsPC-1 cells compared to BxPC-3 and CAPAN-1 cells.

As a marker of double strand DNA breaks (DSBs), γH2AX foci represent a surrogate method for assessing direct DNA damage. Theoretically, only one DSB is required to induce cell death. However as low linear energy transfer (LET) particles, beta particles primarily damage the DNA indirectly through the production of free radicals. The free radicals are responsible for causing DNA breaks, yet the damage only becomes irreparable in the presence of oxygen. In a hypoxic environment, such as PDAC, DNA damage induced by beta-particles is reparable and multiple DSBs are needed to increase the likelihood of cell death. This study found [^177^Lu]Lu-DOTA-C595 could induce a high level of γH2AX foci in PANC-1 and AsPC-1 cells within 1 h of exposure. However, there was a significant reduction in the number of γH2AX foci detected in AsPC-1 cells by 24 h suggesting possible resolution of the DSBs. This effect was not observed in PANC-1 cells. It is possible that the increased surface binding and internalisation of [^177^Lu]Lu-DOTA-C595 on PANC-1 cells led to a higher number of DSBs and cumulative dose effects. Quantification of the number of γH2AX foci induced per cell would improve the interpretation of these results.

To further elucidate the in vitro cytotoxicity of [^177^Lu]Lu-DOTA-C595 and confirm the γH2AX results, clonogenic assays were performed. Clonogenic assays provide a quantitative method to assess all mechanisms of cell death and are the gold standard for assessing the radiation sensitivity of cells. In this study [^177^Lu]Lu-DOTA-C595 induced high levels of cell death in both AsPC-1 and PANC-1 cells. At 500, 750 and 1000 nM of [^177^Lu]Lu-DOTA-C595, PANC-1 cells had significantly lower survival than AsPC-1 cells. This is likely a direct outcome of the higher MUC1-CE expression on PANC-1 cells compared to AsPC-1 leading to a higher cumulative dose and increased cell kill. Qu et al. (2004) identified a similar cell kill between cells with a comparable level of MUC1 expression when using a ^213^Bismuth-DTPA-C595 conjugate. At concentrations of 2000 and 3000 nM of [^177^Lu]Lu-DOTA-C595, no significant differences were identified in the survival of the cell lines. This likely suggests a maximal therapeutic effect had already been induced at these concentrations. The results of the clonogenic assay in [^177^Lu]Lu-DOTA-C595 treated cells confirm the γH2AX findings.

At 100 nM of [^177^Lu]Lu-DOTA-C595, AsPC-1 cells exhibited significantly lower cell survival compared to PANC-1 cells. Cell survival was also similar between cell lines at 250 nM of [^177^Lu]Lu-DOTA-C595. The similar cell survival between AsPC-1 and PANC-1 cell lines at these lower concentrations may be explained by the crossfire effect. As low LET particles, multiple beta-particle interactions with the DNA are required to create sufficient damage for cell kill. The crossfire effect, governed by the beta-particles emitted by Lu-177 (max range 2.5 mm), can enhance cell kill in nearby non-targeted cells by increasing the number of beta-particle interactions. Beta particles are typically estimated to traverse over 100 cell diameters, increasing the dose deposited in the traversed cells. This estimate assumes cells are clustered together; however cells are initially plated individually in clonogenic assays. If we assume the cells are equally distributed within the well and the radionuclide is homogeneously dispersed, then we can estimate there is an average distance of 0.165–0.558 mm and 0.139–0.290 mm between individual PANC-1 and AsPC-1 cells respectively (when accounting for the median cell size and number of seeded cells per well), in the [^177^Lu]Lu-DOTA-C595 treated wells. The shorter average distance between AsPC-1 cells suggests a larger crossfire effect is possible in this population (i.e. more cells traversed by a single beta-particle), leading to a higher number of beta-particle interactions within the traversed cells and an increase in the overall cumulative damage. While the crossfire effect can also increase damage to PANC-1 cells, the higher binding of [^177^Lu]Lu-DOTA-C595 to PANC-1 likely reduced the additional impact of the crossfire, as cells would already receive multiple beta particle interactions from surface-bound or internalised [^177^Lu]Lu-DOTA-C595.

The exact mechanism of [^177^Lu]Lu-DOTA-C595 induced cell death remains unclear. Given the high internalisation efficiency of AsPC-1 and PANC-1 cells, it is reasonable to assume the internalisation process increases the likelihood of cell death. However, the effects of cell membrane damage which may be caused by surface-bound or internalised [^177^Lu]Lu-DOTA-C595 may also contribute to overall cell kill (Zhang et al. 2018). Further work is required to establish the mechanism of [^177^Lu]Lu-DOTA-C595 induced cell death.

Interestingly, unlabeled Lu-177 exhibited a potent effect on both cell lines. In AsPC-1 cell lines, cells treated with unlabeled Lu-177 had significantly lower survival compared to cells treated with equivalent concentrations of [^177^Lu]Lu-DOTA-C595 at 500, 750 and 1000 nM. There was no difference observed between the radioactive treatments at 100 nM in AsPC-1 cells, likely due to the lower radioactivity administered compared to other concentrations. No significant differences were found between the survival of PANC-1 cells treated with unlabeled Lu-177 and [^177^Lu]Lu-DOTA-C595. The similar and, at times, more pronounced cell kill of unlabeled Lu-177 compared to [^177^Lu]Lu-DOTA-C595 may be due to the high levels of radioactivity used which were capable of inducing a therapeutic effect irrespective of binding to MUC1-CE. It is also possible that [^177^Lu]Lu-DOTA-C595 had slower cellular uptake than unlabeled Lu-177 due to the intact monoclonal antibody. This may have reduced the magnitude of effect [^177^Lu]Lu-DOTA-C595 could cause during the incubation time for the clonogenic assays. However, this cannot be confirmed as the study did not assess the cellular internalisation rate of unlabeled Lu-177. In vivo studies will provide an opportunity to assess the difference in binding kinetics of [^177^Lu]Lu-DOTA-C595 and unlabeled Lu-177.

Treatment using unmodified C595 also demonstrated a cytotoxic effect against PDAC cells in this study. Wang et al. (2011) and Shimizu and Imai (2008) have previously reported a similar therapeutic effect in ovarian cancer and oral squamous cell carcinoma cells. However, when assessing the efficacy of unmodified C595 in PDAC cells, Qu et al. (2004) did not identify any significant cytotoxicity. Qu et al. (2004) do not describe the concentrations of C595 applied in their study, thus it may be possible this study used a higher concentration than previously reported, contributing to the increased therapeutic effect. The exact mechanism leading to C595-induced cell death is not yet clear. Previous studies have identified anti-MUC1 immunotherapy can deregulate epidermal growth factor receptor (EGFR) activation and reduce MUC1 signalling (Hisatsune et al. 2011; Wu et al. 2018b). In line with other studies, our results have also demonstrated cytotoxicity of unmodified C595 is dependent on MUC1-CE expression (Wang et al. 2007).

Given the promising preclinical characterisation of [^177^Lu]Lu-DOTA-C595 in PDAC, future research should consider applying [^177^Lu]Lu-DOTA-C595 to other epithelial malignancies with MUC1-CE overexpression such as lung, colon and ovarian adenocarcinoma. Several studies have highlighted the negative association of MUC1-CE overexpression and prognosis in these malignancies, hence anti-MUC1-CE RIT could be utilised against late-stage disease in these cohorts (Wang et al. 2007; Ham et al. 2016; Kasprzak et al. 2018).

To date, this study has only characterised [^177^Lu]Lu-DOTA-C595 in in vitro experiments. While these are valid experiments for assessing the mechanism of cellular binding, internalisation and cytotoxicity, we are unable to assess the systemic effects of [^177^Lu]Lu-DOTA-C595 on normal cells and overall tumour burdens. In vivo studies are required to better assess all range of effects. Further, despite identifying [^177^Lu]Lu-DOTA-C595 can induce a significant effect on PDAC cells, alpha-emitting radionuclides can produce superior radiobiological effects and may enhance overall toxicity. Alpha particles have a higher LET and induce dense double-strand DNA breaks through direct ionisation events. Replacing Lu-177 with an alpha-emitting radionuclide such as Actinium-225 may improve the cytotoxic effect observed by [^177^Lu]Lu-DOTA-C595 in this study.

## Conclusion

In vitro characterisation of new radiopharmaceuticals, particularly therapeutic radioimmunoconjugates, is integral in the preclinical development phase to prevent unnecessary harm to animals and to gain invaluable insight into the in vitro behavior of the agent of interest. This study characterised [^177^Lu]Lu-DOTA-C595 against MUC1-CE expressing PDAC cells. It was demonstrated [^177^Lu]Lu-DOTA-C595 bound to cells with high MUC1-CE expression and was rapidly internalised. A high number of γH2AX foci were induced to suggest cumulative effects of indirect DNA damage and clonogenic assays further highlighted the cytotoxicity of [^177^Lu]Lu-DOTA-C595. The study has shown promising results warranting further studies in animals to understand the in vivo characteristics of [^177^Lu]Lu-DOTA-C595 in targeting of PDAC and other epithelial malignancies with MUC1-CE overexpression.

## Data Availability

Data is available upon reasonable request to the corresponding author.
